# Low 25-hydroxyvitamin D_2_ and 25-hydroxyvitamin D_3_ levels are independently associated with macroalbuminuria, but not with retinopathy and macrovascular disease in type 1 diabetes: the EURODIAB prospective complications study

**DOI:** 10.1186/s12933-015-0231-2

**Published:** 2015-05-30

**Authors:** Lian Engelen, Casper G. Schalkwijk, Simone J. P. M. Eussen, Jean L. J. M. Scheijen, Sabita S. Soedamah-Muthu, Nish Chaturvedi, John H. Fuller, Coen D. A. Stehouwer

**Affiliations:** Department of Internal Medicine, Maastricht University Medical Centre, Universiteitssingel 50, 6200 MD Maastricht, The Netherlands; CARIM School for Cardiovascular Diseases, Maastricht University Medical Centre, Maastricht, The Netherlands; Top Institute Food and Nutrition, Wageningen, The Netherlands; CAPHRI School for Public Health and Primary Care, Maastricht University Medical Centre, Maastricht, The Netherlands; Department of Epidemiology, Maastricht University, Maastricht, The Netherlands; Department of Global Public Health and Primary Care, University of Bergen, Bergen, Norway; Division of Human Nutrition, Wageningen University, Wageningen, The Netherlands; International Centre for Circulatory Health, National Heart and Lung Institute, Imperial College London, London, UK; Department of Epidemiology and Public Health, University College, London, UK

**Keywords:** 25-hydroxyvitamin D_2_, 25-hydroxyvitamin D_3_, Albuminuria, Cardiovascular disease, Microvascular complications, Retinopathy, Type 1 diabetes, Vitamin D

## Abstract

**Background:**

Low circulating levels of total vitamin D [25(OH)D] and 25(OH)D_3_ have been associated with vascular complications in few studies on individuals with type 1 diabetes. However, these measures are affected by UV light exposure. Circulating 25(OH)D_2_, however, solely represents dietary intake of vitamin D_2_, but its association with complications of diabetes is currently unknown. We investigated the associations between 25(OH)D_2_ and 25(OH)D_3_ and the prevalence of albuminuria, retinopathy and cardiovascular disease (CVD) in individuals with type 1 diabetes.

**Methods:**

We measured circulating 25(OH)D_2_ and 25(OH)D_3_ in 532 individuals (40 ± 10 years old, 51 % men) with type 1 diabetes who participated in the EURODIAB Prospective Complications Study. Cross-sectional associations of 25(OH)D_2_ and 25(OH)D_3_ with albuminuria, retinopathy and CVD were assessed with multiple logistic regression analyses adjusted for age, sex, season, BMI, smoking, HbA_1c_, total-HDL-cholesterol-ratio, systolic blood pressure, antihypertensive medication, eGFR, physical activity, alcohol intake, albuminuria, retinopathy and CVD, as appropriate.

**Results:**

Fully adjusted models revealed that 1 nmol/L higher 25(OH)D_2_ and 10 nmol/L higher 25(OH)D_3_ were associated with lower prevalence of macroalbuminuria with ORs (95 % CI) of 0.56 (0.43;0.74) and 0.82 (0.72;0.94), respectively. These vitamin D species were not independently associated with microalbuminuria, non-proliferative and proliferative retinopathy or CVD.

**Conclusions:**

In individuals with type 1 diabetes, both higher 25(OH)D_2_ and 25(OH)D_3_ are associated with a lower prevalence of macroalbuminuria, but not of retinopathy and CVD. Prospective studies are needed to further examine the associations between 25(OH)D_2_ and 25(OH)D_3_ and the development of microvascular complications and CVD in type 1 diabetes.

**Electronic supplementary material:**

The online version of this article (doi:10.1186/s12933-015-0231-2) contains supplementary material, which is available to authorized users.

## Background

Vitamin D plays an important role in human health, in particular in bone metabolism [1]. In addition, vitamin D deficiency has been shown to be involved in several pathophysiological processes, such as inflammation [2–4], endothelial dysfunction [5, 6] and up-regulation of the renin-angiotensin-aldosterone system (RAAS) [7], that have been associated with the development of complications of type 1 diabetes [8–10]. In line with this, few observational cohort studies in individuals with type 1 diabetes have shown associations between low 25-hydroxyvitamin D [25(OH)D, which is the sum of 25(OH)D_2_ and 25(OH)D_3_] [11, 12] and low 25(OH)D_3_ [13] on the one hand and microvascular complications [11, 12] and mortality [13] on the other.

Although 25(OH)D_3_ can be produced after consumption of vitamin D_3_-containing foods such as meat, egg yolk and fatty fish, vitamin D_3_-fortified foods and vitamin D_3_ supplements, it is mainly produced when skin is exposed to UV light [1]. As any disease state is likely to reduce physical activity and thereby UV light exposure and 25(OH)D_3_ levels, reverse causality is an important limitation of observational studies on the associations between total 25(OH)D or 25(OH)D_3_ and disease [14]. In contrast, circulating 25(OH)D_2_ is not directly influenced by exposure to UV light and thus solely represents dietary intake of vitamin D_2_, mainly from shiitake mushrooms, egg yolk and potentially other foods [1], vitamin D_2_-fortified foods and from vitamin D_2_ supplements [1, 15]. It is therefore of interest to specifically investigate and compare associations between the two 25(OH)D metabolites – 25(OH)D_2_ and 25(OH)D_3_ – and vascular disease. So far no studies have been published on the associations between circulating 25(OH)D_2_ and microvascular complications and cardiovascular disease (CVD) in individuals with type 1 diabetes.

We therefore aimed to investigate the associations between both circulating 25(OH)D_2_ and 25(OH)D_3_ and the prevalence of microalbuminuria, macroalbuminuria, and non-proliferative and proliferative retinopathy and CVD in a European cohort of individuals with type 1 diabetes.

## Methods

### Study population

We used data from the EURODIAB Prospective Complications Study, a European based prospective cohort study [16, 17]. In brief, baseline investigations were performed between 1989 and 1991 on 3,250 patients with type 1 diabetes, defined as clinical diagnosis made before the age of 36, and needing continuous insulin therapy within one year of diagnosis. Patients aged 15–60 years were recruited from 31 centres in 16 European countries. Sample selection was stratified by sex, age group and duration of diabetes to ensure sufficient representation in all categories. These patients were invited for a follow-up examination 7–9 years after the baseline examinations. Of the 3,250 included patients, 1,880 (58 %) returned for re-examination. At follow-up, a cross-sectional nested case–control study of markers of inflammation and endothelial dysfunction and their associations with complications was performed in a subset of patients (*n* = 543). Cases were selected as those with the greatest vascular complication burden as possible (i.e., individuals with CVD or proliferative retinopathy or macroalbuminuria and individuals with microalbuminuria and some degree of retinopathy, *n* = 348) and controls were selected as those who were completely free of any complications (i.e., with no evidence of CVD, albuminuria and retinopathy; *n* = 195) [18]. The present study includes 532 of these patients from the cross-sectional case–control study at follow-up in whom plasma samples were available for determination of vitamin D.

Ethics committee approval conformed to the Declaration of Helsinki was obtained at each centre and all participants provided written informed consent.

### Measurement of 25-hydroxyvitamin D_2_ and 25-hydroxyvitamin D_3_

Concentrations of 25(OH)D_2_ and 25(OH)D_3_ were determined with the use of ultra-performance liquid chromatography (UPLC) tandem mass spectrometry (MS) in plasma samples that were stored at −80 °C until analyses.

Two hundred μL plasma was mixed with 200 μL [d_3_]-25(OH)D_3_ (50 nmol/L ethanol) and 20 μL perchloric acid (50 %, v/v), 1600 μL hexane was added and samples were centrifuged for 10 min at 14000 rpm at a temperature of 4 °C. The supernatant was dried under a stream of nitrogen and derivatised with 100 μL 4-phenyl-1,2,4-triazoline-3,5-dione (PTAD, 0,1 mg/mL ethylacetate) for 30 min, both at room temperature. Excess PTAD was removed with 400 μL ethanol and the supernatant was then again dried under a stream of nitrogen. The residue was dissolved in 50 μL acetonitrile and 5 μL was injected for analysis by UPLC (Acquity UPLC, Waters, Milford, USA) and detection by tandem MS in ESI positive multiple reaction monitoring (MRM) mode using a Xevo TQ MS (Waters, Milford, USA). Derivatives were separated on a reversed-phase C18 column (Acquity UPLC BEH C18, 100 x 2.1 mm, 1.7 μm) with a binary gradient of 5 mmol/L formic acid and acetonitril at a flow rate of 500 μL/min. The injection volume was 5 μL and column temperature was set at 35 °C. Quantification of 25(OH)D_2_ and 25(OH)D_3_ was performed by calculating the peak area ratio of each unlabeled peak area to the peak area of the internal standard [d_3_]-25(OH)D_3_. The MRM transitions for 25(OH)D_2_ and 25(OH)D_3_ were 570.3 > 298.1 and 558.2 > 298.1, respectively. The MRM transitions for the internal standard [d_3_]-25(OH)D_3_ was 561.2 > 301.1. Electrospray ionization was done at a capillary voltage of 1.5 kV, a source temperature of 150 °C and a desolvation temperature of 600 °C.

For validation purposes, linearity was determined by adding standard solution of 25(OH)D_2_ and 25(OH)D_3_ to water and plasma. A six-point calibration curve was prepared for 25(OH)D_2_ (0–79.6 nmol/L) and 25(OH)D_3_ (0–16.2 nmol/L). The peak area ratio of 25(OH)D_2_ and 25(OH)D_3_ multiplied by the concentration of the internal standard were plotted as a function of the concentration. Calibration curves for 25(OH)D_2_ and 25(OH)D_3_ were linear over the described ranges (*r*^*2*^ > 0.99) in both water and plasma. Mean slopes (response factors) for 25(OH)D_2_ and 25(OH)D_3_ were 0.9594 [coefficient of variation (CV): 6.1 %] and 0.6212 (CV: 14.9 %), respectively. Inter- and intra-assay CVs for concentrations of 25(OH)D2 were 4.6 % and 3.1 % and for concentrations of 25(OH)D_2_ were 8.9 % and 9.1 %, respectively.

### Microvascular complications

The albumin excretion rate (AER) was measured from duplicate 24-h urine collections using an immunoturbidimetric method that included goat anti-human albumin antisera (Sanofi Diagnostics Pasteur, Chaska, MN, USA) and human serum albumin standards (ORHA 20/21 grade HSA; Behring Diagnostics, Hoechst UK, Hounslow, UK) [19]. Microalbuminuria and macroalbuminuria were defined as AER 20–200 μg/min and >200 μg/min, respectively.

Retinopathy was centrally assessed from retinal photographs by a trained reader of colour retinal photographs using a system of 45° field grading standards for the assessment of retinopathy that was developed for the EURODIAB Prospective Complications Study. Non-proliferative retinopathy was defined as the presence of one or more microaneurysms, haemorrhages and/or hard exudates. Proliferative retinopathy was defined as any new vessel, fibrous proliferation, pre-retinal haemorrhage, vitreous haemorrhage or photocoagulation scar [20].

### Cardiovascular disease

CVD was defined as a positive medical history of a cardiovascular event, including myocardial infarction, angina pectoris, coronary artery bypass graft and/or stroke, and/or ischaemic changes on a centrally Minnesota-coded ECG [17].

### Covariables

Weight and height were measured with indoor clothing without shoes and body mass index (BMI) was calculated as weight divided by height squared. Smoking habits were ascertained by questionnaire and individuals were categorised into never smokers, ex-smokers and current smokers. Glycated haemoglobin (HbA_1c_) was measured by a latex enhanced turbidimetric immunoassay. Cholesterol levels were measured with colorimetric tests and HDL cholesterol was measured directly. Blood pressure was recorded twice with a random zero sphygmomanometer (Hawskley, Lancing, UK). The glomerular filtration rate (GFR) was estimated using the Chronic Kidney Disease Epidemiology Collaboration (CKD-EPI) equation [21]. Self-reported physical activity was assessed by a questionnaire [22]. The weekly metabolic equivalent (MET-h) corresponds to the weekly amount of time spent in each sporting activity multiplied by the corresponding MET value. Physical activity was then categorized into none, low (≤sex-specific median) and high (>sex-specific median). Alcohol intake was assessed using the general questionnaire and determined by multiplying the weekly intake of each alcoholic beverage by its ethanol content [23]. Alcohol intake was categorized into abstainers, low (≤70 g/week) and high (>70 g/week). We measured plasma levels of C-reactive protein (CRP) with a highly sensitive in-house ELISA [24] and plasma levels of IL-6 and TNF-α with commercially available ELISA kits (R&D Systems, Oxon, UK) [24], as markers of low-grade inflammation. Soluble vascular cell adhesion molecule-1 (sVCAM-1) and soluble E-selectin (sE-selectin) were measured in duplicate by sandwich enzyme immunoassays (R&D Systems, Oxon, UK), as markers of endothelial dysfunction.

### Statistical analyses

All statistical analyses were carried out using the Statistical Package for the Social Sciences, version 20.0 (SPSS IBM Corporation, Armonk, NY, USA), unless specified otherwise. General characteristics were compared between tertiles of 25(OH)D_2_ and, to account for seasonal variation due to UV light exposure, *month-specific* tertiles of 25(OH)D_3_ with the use of ANOVA or *χ*^2^ tests for continuous and categorical data, respectively. Seven percent (37 individuals) of the total population had missing values for one (*n* = 30) or more (*n* = 7) of the covariates. The percentage of missing values per variable varied from 0.2 % (physical activity) to 2.6 % (TNF-α). We used multiple imputation chained equations to impute those missing values rather than perform complete case analyses in order to decrease bias and increase the power of the analyses [25].

We used multinomial logistic regression analyses to investigate the associations between 25(OH)D_2_ and 25(OH)D_3_ levels and the prevalence of microalbuminuria and macroalbuminuria, and of non-proliferative and proliferative retinopathy, respectively. Binary logistic regression analyses were performed to investigate the associations between 25(OH)D_2_ and 25(OH)D_3_ levels and the prevalence of CVD. All analyses were adjusted for age and sex [and, in the case of 25(OH)D_3_, season of examination] (model 1) and additionally for BMI, smoking, HbA_1c_, total-HDL-cholesterol-ratio, systolic blood pressure, use of antihypertensive medication, eGFR, physical activity, alcohol intake, and for prevalent CVD, albuminuria and retinopathy, as appropriate (model 2). We investigated these associations both with 25(OH)D_2_ tertiles and month-specific 25(OH)D_3_ tertiles, and with 25(OH)D_2_ and 25(OH)D_3_ levels as continuous variables [additionally adjusting for season of examination in the case of 25(OH)D_3_].

Decreased kidney function may impair the conversion of 25(OH)D into its active form 1,25(OH)_2_D [1]. We therefore tested for interaction with eGFR, on the multiplicative scale by adding product terms between eGFR and 25(OH)D_2_ and 25(OH)D_3_ levels to the fully adjusted models, and on the additive scale by calculating the relative excess risk due to interaction [26].

Lastly, linear regression analyses were conducted to investigate the associations between 25(OH)D_2_ and 25(OH)D_3_ and markers of low-grade inflammation (both individually and comprised in an averaged Z-score of CRP, IL-6 and TNF-α) and endothelial function (both individually and comprised in an averaged Z-score of sVCAM-1 and sE-selectin-1) [27]. Furthermore, in the case of significant associations between 25(OH)D_2_ and 25(OH)D_3_ and vascular complications, we additionally adjusted these associations for the low-grade inflammation and endothelial dysfunction scores to investigate to what extent these pathophysiological processes explained (i.e., attenuated) these associations.

## Results

In the total population, median (IQR) plasma 25(OH)D_2_ and 25(OH)D_3_ concentrations were 1.5 (1.0-2.4) nmol/L and 51 (33–74) nmol/L, respectively. Plasma levels of 25(OH)D_2_ and 25(OH)D_3_ were not correlated (*r*_*S*_ = −0.04, *p* = 0.363).

**Fig. 1:**
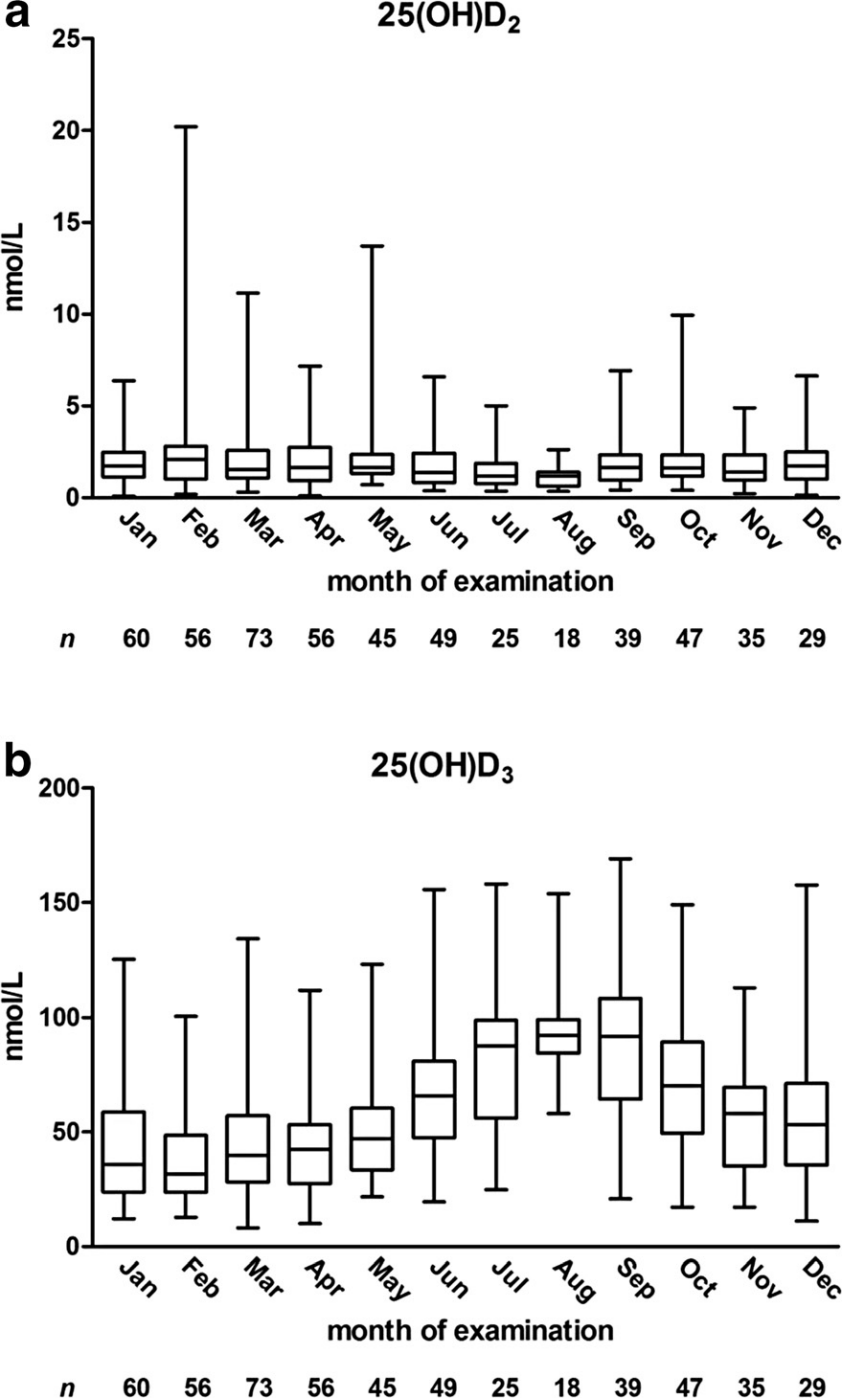
Concentration of 25(OH)D_2_ (**a**) and 25(OH)D_3_ (**b**) according to month of examination. Boxes indicate medians and interquartile ranges and whiskers represent the minimum and maximum values

Figure 1 shows plasma levels of 25(OH)D_2_ and 25(OH)D_3_ across months of examination. Levels of 25(OH)D_3_, which are dependent on sunlight exposure, differed according to season of examination, whereas levels of 25(OH)D_2_ did not. We calculated month-specific tertiles of 25(OH)D_3_ to account for the variation in 25(OH)D_3_ between seasons and used these in all further analyses with tertiles of 25(OH)D_3_. Additional file 1: Figure S1 (supplemental material) shows plasma levels of 25(OH)D_2_ and 25(OH)D_3_ according to latitude of the study centre. We found no evidence for decreasing levels of 25(OH)D_2_ or 25(OH)D_3_ with increasing latitude.

Eighty-three and 124 individuals had microalbuminuria and macroalbuminuria, respectively; 146 and 152 individuals had non-proliferative and proliferative retinopathy, respectively, and 131 individuals had CVD.

### Population characteristics

Table 1 shows the general characteristics of the EURODIAB Prospective Complications Study population according to tertiles of 25(OH)D_2_ and month-specific tertiles of 25(OH)D_3_.

**Table 1:** General characteristics of the study population (*n* = 532) according to tertiles of 25-hydroxyvitamin D_2_ and month-specific tertiles of 25-hydroxyvitamin D_3_

	25-hydroxyvitamin D_2_			25-hydroxyvitamin D_3_		
	T1 (*n* = 177, 0.08-1.19 nmol/L)	T2 (*n* = 178, 1.20-2.09 nmol/L)	T3 (*n* = 177, 2.10-20.2 nmol/L)	T1 (*n* = 177, 8–86 nmol/L)^d^	T2 (*n* = 177, 27–100 nmol/L)^d^	T3 (*n* = 178, 43–169 nmol/L)^d^
Age (years)	40.5 ± 10.5	39.0 ± 9.8	39.2 ± 10.0	40.9 ± 10.4	39.5 ± 10.3	38.2 ± 9.4
Male sex (%)	51	47	55	53	52	49
Body mass index (kg/m^2^)	24.5 ± 3.4	24.7 ± 3.1	24.3 ± 3.3	24.7 ± 3.7	24.5 ± 3.1	24.2 ± 2.8
HbA_1c_ (%) (mmol/mol)	8.7 ± 1.7 (72 ± 19)	8.5 ± 1.7 (69 ± 19)	8.5 ± 1.5 (69 ± 16)	9.0 ± 1.8 (75 ± 20)	8.4 ± 1.5 (68 ± 16)	8.2 ± 1.5 (66 ± 16)
Duration of diabetes (years)	22.9 ± 9.0	20.5 ± 9.7	20.9 ± 9.3	22.8 ± 9.2	22.2 ± 9.9	19.4 ± 8.8
Total cholesterol (mmol/L)	5.4 ± 1.4	5.4 ± 1.1	5.1 ± 1.1	5.4 ± 1.3	5.2 ± 1.1	5.3 ± 1.2
LDL cholesterol (mmol/L)	3.2 ± 1.2	3.1 ± 1.0	3.2 ± 1.0	3.2 ± 1.1	3.1 ± 1.0	3.2 ± 1.1
HDL cholesterol (mmol/L)	1.6 ± 0.5	1.6 ± 0.4	1.6 ± 0.4	1.6 ± 0.4	1.6 ± 0.4	1.7 ± 0.4
Total-HDL-cholesterol-ratio	3.7 ± 1.3	3.5 ± 1.4	3.3 ± 1.0	3.7 ± 1.5	3.4 ± 1.1	3.3 ± 1.1
Triacylglycerols (mmol/L)	1.0 (0.8-1.4)	1.0 (0.7-1.4)	0.9 (0.7-1.2)	1.0 (0.7-1.5)	1.0 (0.8-1.4)	0.9 (0.7-1.2)
Smoking (never/ex/current, %)	39/32/29	39/32/29	42/24/34	37/28/35	42/30/28	41/30/29
Physical activity (0/≤median^a^/>median, %)	68/17/15	59/20/21	60/21/19	74/14/12	63/20/17	50/25/25
Alcohol intake (0/≤70 g per wk/>70 g per wk, %)	16/53/31	19/53/28	10/57/33	21/53/26	13/56/31	10/55/35
Systolic blood pressure (mmHg)	127 ± 22	121 ± 19	120 ± 18	125 ± 21	121 ± 20	123 ± 18
Diastolic blood pressure (mmHg)	76 ± 11	75 ± 12	74 ± 11	76 ± 12	73 ± 11	76 ± 11
Use of antihypertensive medication (%)	45	31	19	36	33	26
Use of hormone replacement therapy (% women)	14	11	10	11	8	15
Use of oral contraceptive therapy (% women)	39	42	47	27	49	50
Estimated glomerular filtration rate (mL/min)	97 (77–109)	103 (88–114)	104 (91–113)	102 (82–112)	102 (86–112)	101 (89–112)
C-reactive protein (mg/L)	1.1 (0.5-2.7)	1.0 (0.4-2.5)	1.1 (0.4-2.4)	1.4 (0.5-3.5)	0.9 (0.4-2.3)	0.9 (0.4-2.4)
Interleukin-6 (pg/mL)	2.1 (1.5-4.0)	1.8 (1.1-3.0)	1.8 (1.2-3.1)	2.3 (1.5-4.4)	1.9 (1.2-3.0)	1.6 (1.1-2.8)
Tumour necrosis factor-α (pg/mL)	3.2 (2.3-4.6)	2.7 (2.0-3.3)	2.6 (2.0-3.2)	3.1 (2.2-4.3)	2.7 (2.1-3.5)	2.7 (2.1-3.4)
Low-grade inflammation score (SD)^b^	0.27 ± 1.00	−0.13 ± 0.99	−0.14 ± 0.97	0.26 ± 1.10	−0.11 ± 0.87	−0.14 ± 0.97
Soluble E-selectin (ng/mL)	36 ± 20	32 ± 11	35 ± 12	36 ± 16	35 ± 16	32 ± 13
Soluble vascular cell adhesion molecule-1 (ng/mL)	451 ± 174	391 ± 97	387 ± 98	438 ± 169	399 ± 106	393 ± 103
Endothelial dysfunction score (SD)^c^	0.30 ± 1.32	−0.22 ± 0.74	−0.08 ± 0.76	0.21 ± 1.15	−0.04 ± 0.97	−0.17 ± 0.82
Albuminuria (normo/micro/macro, %)	45/13/42	65/17/18	73/17/10	52/14/34	58/20/22	74/13/13
Retinopathy (none/non-proliferative/proliferative, %)	34/30/36	49/27/24	49/25/26	34/33/33	44/28/28	54/21/25
Cardiovascular disease (%)	21	29	24	28	27	20
25-hydroxyvitamin D_2_ (nmol/L)	0.8 (0.6-1.0)	1.5 (1.4-1.8)	2.8 (2.4-3.9)	1.4 (0.9-2.5)	1.6 (1.1-2.4)	1.6 (1.1-2.4)
25-hydroxyvitamin D_3_ (nmol/L)	46 (28–73)	58 (37–75)	49 (33–74)	27 (21–38)	48 (39–67)	77 (62–98)

Data are presented as means ± SD, medians (interquartile range) or percentages, as appropriate. ^a^sex-specific medians for physical activity were 11.3 MET-h/week for men and 6.8 MET-h/week for women; ^b^the low-grade inflammation score was calculated as the mean Z-score of ln-transformed values of C-reactive protein, interleukin-6 and tumour necrosis factor-α; ^c^the endothelial dysfunction score was calculated as the mean Z-score of soluble E-selectin and soluble vascular cell adhesion molecule-1; ^d^ranges of 25-hydroxyvitamin D_3_ concentrations overlap between tertiles as tertiles were defined month-specifically (see Methods section)

Individuals with higher 25(OH)D_2_ and those with higher month-specific 25(OH)D_3_ concentrations had on average lower values of total-HDL-cholesterol-ratio and markers of low-grade inflammation and endothelial dysfunction compared to those with lower vitamin D concentrations. In addition, they less often used antihypertensive medication and less often had albuminuria and retinopathy.

Finally, those with higher 25(OH)D_2_ concentrations had on average lower systolic blood pressure, but higher eGFR, whereas individuals with higher month-specific 25(OH)D_3_ were on average somewhat younger and had lower HbA_1c_ and duration of diabetes, but higher physical activity and alcohol intake (Table 1).

### Associations between 25-hydroxyvitamin D_2_ and albuminuria, retinopathy and cardiovascular disease

Fully adjusted continuous analyses showed that 1 nmol/L higher 25(OH)D_2_ was non-significantly associated with lower prevalence of microalbuminuria, with an OR (95 % CI) of 0.85 (0.70;1.04), and significantly with macroalbuminuria [0.56 (0.43;0.74)]. Associations between 25(OH)D_2_ and non-proliferative retinopathy [1.00 (0.85;1.16)], proliferative retinopathy [1.08 (0.93;1.25)] and CVD [1.03 (0.90;1.16)] were not significant (Table 2, model 2). Associations between tertiles of 25(OH)D_2_ and microvascular complications showed comparable results. However, comparing tertile 2 (T2) vs. T1 of 25(OH)D_2,_ 25(OH)D_2_ was associated with higher prevalence of CVD [2.22 (1.23;4.01)], whereas T3 vs. T1 was not [1.64 (0.89;3.05)] (Additional file 1: Table S1, model 2).

**Table 2:** Associations between 25-hydroxyvitamin D_2_ levels and prevalent albuminuria, retinopathy and cardiovascular disease

		25-hydroxyvitamin D_2_ per 1 nmol/L		
	Model	OR	95 % CI	*p*
Microalbuminuria (*n* = 83)	1	0.94	0.81; 1.09	0.42
	2	0.85	0.70; 1.04	0.11
	3	0.86	0.71; 1.05	0.14
	4	0.85	0.70; 1.03	0.09
Macroalbuminuria (*n* = 124)	1	0.45	0.35; 0.60	<0.001
	2	0.56	0.43; 0.74	<0.001
	3	0.57	0.43; 0.75	<0.001
	4	0.56	0.42; 0.74	<0.001
Non-proliferative retinopathy (*n* = 146)	1	0.91	0.80; 1.03	0.15
	2	1.00	0.85; 1.16	0.95
Proliferative retinopathy (*n* = 152)	1	0.88	0.77; 1.00	0.06
	2	1.08	0.93; 1.25	0.33
CVD (*n* = 131)	1	1.02	0.92; 1.14	0.70
	2	1.03	0.90; 1.16	0.70

*OR*, odds ratio indicates the odds of prevalent albuminuria, retinopathy and CVD, per 1 nmol/L higher 25-hydroxyvitamin D_2_. Model 1: adjusted for age and sex; Model 2: model 1 + BMI, smoking (never, ex, current), HbA_1c_, total-HDL-cholesterol-ratio, systolic blood pressure, use of antihypertensive medication, eGFR, physical activity (0, ≤sex-specific median, >sex-specific median), alcohol intake (0, ≤70 g/wk, >70 g/wk), and prevalent CVD, albuminuria and retinopathy, as appropriate; Model 3: model 2 + low-grade inflammation score; Model 4: model 2 + endothelial dysfunction score

### Associations between 25-hydroxyvitamin D_3_ and albuminuria, retinopathy and cardiovascular disease

Fully adjusted continuous analyses showed that 10 nmol/L higher 25(OH)D_3_ was associated with lower prevalence of macroalbuminuria [0.82 (0.72;0.94)], but not with microalbuminuria [0.93 (0.83;1.05)], non-proliferative retinopathy [0.98 (0.87;1.09)], proliferative retinopathy [1.01 (0.90;1.13)] and CVD [1.02 (0.93;1.12)] (Table 3, model 2). Associations between month-specific tertiles of 25(OH)D_3_ and microvascular complications and CVD showed comparable results (Additional file 1: Table S2, model 2).

**Table 3:** Associations between 25-hydroxyvitamin D_3_ levels and prevalent albuminuria, retinopathy and CVD

		25-hydroxyvitamin D_3_ per 10 nmol/L		
	Model	OR	95 % CI	*p*
Microalbuminuria (*n* = 83)	1	0.93	0.84; 1.03	0.17
	2	0.93	0.83; 1.05	0.26
	3	0.94	0.83; 1.06	0.30
	4	0.94	0.83; 1.06	0.30
Macroalbuminuria (*n* = 124)	1	0.80	0.72; 0.88	<0.001
	2	0.82	0.72; 0.94	0.003
	3	0.82	0.72; 0.94	0.005
	4	0.82	0.72; 0.94	0.005
Non-proliferative retinopathy (*n* = 146)	1	0.90	0.82; 0.99	0.022
	2	0.98	0.87; 1.09	0.66
Proliferative retinopathy (*n* = 152)	1	0.90	0.83; 0.98	0.018
	2	1.01	0.90; 1.13	0.87
CVD (*n* = 131)	1	0.99	0.91; 1.08	0.84
	2	1.02	0.93; 1.12	0.66

*OR*, odds ratio indicates the odds of prevalent albuminuria, retinopathy and CVD per 10 nmol/L higher 25-hydroxyvitamin D_3_. Model 1: adjusted for age, sex and season; Model 2: model 1 + BMI, smoking (never, ex, current), HbA_1c_, total-HDL-cholesterol-ratio, systolic blood pressure, use of antihypertensive medication, eGFR, physical activity (0, ≤sex-specific median, >sex-specific median), alcohol intake (0, ≤70 g/wk, >70 g/wk), and prevalent albuminuria, retinopathy and CVD, as appropriate; Model 3: model 2 + low-grade inflammation score; Model 4: model 2 + endothelial dysfunction score

### Additional analyses

Additional adjustment for hormone replacement therapy and/or oral contraceptive therapy did not materially change the results (data not shown).

We found no consistent significant additive or multiplicative interactions between 25(OH)D_2_ or 25(OH)D_3_ and eGFR in the associations with microvascular complications and CVD (data not shown).

Additional file 1: Table S3 and S4 show the associations between 25(OH)D_2_ and 25(OH)D_3_ levels, respectively, and markers of low-grade inflammation and endothelial dysfunction.

Fully adjusted continuous analyses showed that 1 nmol/L higher 25(OH)D_2_ was not significantly associated with lower scores of low-grade inflammation and endothelial dysfunction. However, comparing T2 vs. T1 of 25(OH)D_2_ [−0.239 SD (−0.420;-0.058)] as well as T3 vs. T1 [−0.190 (−0.371;-0.009)], 25(OH)D_2_ was associated with lower low-grade inflammation. In addition, both 25(OH)D_2_ T2 vs. T1 [−0.363 (−0.557;-0.170)] and T3 vs. T1 [−0.234 (−0.426;-0.043)] were associated with lower endothelial dysfunction (Table S3, model 2).

Fully adjusted continuous analyses showed that 10 nmol/L higher 25(OH)D_3_ was inversely associated with lower endothelial dysfunction [−0.040 (−0.073;-0.007)], but not low-grade inflammation [−0.019 (−0.049;0.011)]. In addition, comparing month-specific T2 vs. T1 of 25(OH)D_3_ [−0.206 (−0.387;-0.024)], but not T3 vs. T1 [−0.131 (−0.317;0.056)], 25(OH)D_3_, was associated with lower low-grade inflammation and month-specific 25(OH)D_3_ T3 vs. T1 [−0.215 (−0.416;-0.015)], but not T2 vs. T1 [−0.149 (−0.343;0.046)], was associated with lower endothelial dysfunction (Additional file 1: Table S4, model 2).

We additionally adjusted the associations between 25(OH)D_2_ and 25(OH)D_3_ and microalbuminuria and macroalbuminuria for the low-grade inflammation and endothelial dysfunction scores and found that these associations did not materially change after adjustment for low-grade inflammation and endothelial dysfunction (Tables 2 and 3, model 3 and 4, respectively).

## Discussion

The present study showed that in individuals with type 1 diabetes, higher circulating 25(OH)D_2_ as well as 25(OH)D_3_ were associated with a lower prevalence of macroalbuminuria, but not with retinopathy and CVD. These associations were independent of age, sex, cardiovascular risk factors and markers of low-grade inflammation and endothelial dysfunction and did not differ between individuals with an eGFR below and above 90 mL/min.

The inverse associations between 25(OH)D_2_ and 25(OH)D_3_ and macroalbuminuria are in line with a previous prospective study in individuals with type 1 diabetes showing an independent and inverse association between total 25(OH)D and microalbuminuria [11]. Our results extend these previously published results by showing not only associations between 25(OH)D_3_ and macroalbuminuria, but also similar associations between 25(OH)D_2_ and macroalbuminuria. Although vitamin D_2_ and D_3_ exhibit identical sets of biological responses around the body, primarily through the same vitamin D receptor mediated regulation of gene expression [28], reverse causation due to physical activity and influence of seasonal variation were avoided in these analyses as circulating 25(OH)D_2_ is not influenced by exposure to UV light. These findings therefore support the association between vitamin D and albuminuria. Other cross-sectional [12] and prospective [13] studies showed no associations between total 25(OH)D [12] or 25(OH)D_3_ [13] and albuminuria in individuals with type 1 diabetes. Discrepancies between the current findings and these previous findings may be explained by the lower numbers of albuminuria cases in the previous than in the current study. Also, in the study by Joergensen et al. [13], 25(OH)D_3_ was categorized and vitamin D deficiency was accordingly defined as less than or equal to the lowest 10^th^ percentile of 25(OH)D_3_ concentration. However, seasonal variation was not adjusted for, which could have resulted in misclassification of vitamin D deficient patients (i.e., possibly incorrectly classifying more patients as vitamin D deficient in winter than in summer), thereby masking potential associations. The single study that showed no association between total 25(OH)D and albuminuria in the general population [29] was also not adjusted for seasonal variation, which may explain the lack of association. In addition, independent and inverse associations between total 25(OH)D and albuminuria have been found in individuals with type 2 diabetes [30, 31] and in the general population [32–34], which further support our current findings in individuals with type 1 diabetes.

We found no independent associations between 25(OH)D_2_ and 25(OH)D_3_ and retinopathy. The significant age- and sex-adjusted associations between both 25(OH)D_2_ and 25(OH)D_3_ and proliferative retinopathy attenuated and became non-significant after adjustment for other potential confounders (Table 2 and 3), in particular after adjustment for albuminuria status (data not shown). Associations with albuminuria and retinopathy are difficult to tease apart as these disease states strongly correlate with each other. We therefore cannot exclude any direct associations between both 25(OH)D_2_ and 25(OH)D_3_ and proliferative retinopathy. In a previous cross-sectional study on individuals with type 1 diabetes, total 25(OH)D has been associated with a lower prevalence of retinopathy [12], a finding that could not be confirmed in another prospective study however [13]. In individuals with type 2 diabetes results have also been conflicting as, similarly, both inverse [31, 35] and no independent associations [35, 36] have been described between total 25(OH)D and prevalence of retinopathy.

The present study did not show a continuous inverse association between 25(OH)D_2_ and 25(OH)D_3_ and CVD. Only individuals in the 2^nd^ tertile, but not those in the 3^rd^ tertile of 25(OH)D_2,_ compared to those in the 1^st^ tertile, had a higher prevalence of CVD, which may reflect a chance finding. In the general population, the *inverse* association between 25(OH)D and CVD has been extensively described in meta-analyses [as summarized in [37]], whereas no studies have been published on the associations between total 25(OH)D, 25(OH)D_2_ or 25(OH)D_3_ and prevalent or incident CVD in type 1 diabetes. Larger, prospective studies are needed to further examine the associations between total 25(OH)D, 25(OH)D_2_ and 25(OH)D_3_ and the development of retinopathy and CVD in type 1 diabetes.

### Potential mechanisms

Vitamin D receptors are present on a large variety of cell types, including most immune cells and vascular endothelial cells [38]. Overall, 1,25(OH)_2_D controls inflammatory and immune responses, keeping them within physiological boundaries [39]. In addition, vitamin D improves endothelial cell function, regulating endothelial cell-dependent vasodilation [5, 6]. Furthermore, the processes of (low-grade) inflammation and endothelial dysfunction have been associated with vascular complications in individuals with type 1 diabetes [8, 9] and we therefore hypothesized that the associations between 25(OH)D_2_ and 25(OH)D_3_ could be explained by these pathophysiological processes. In the current study we indeed found associations between both 25(OH)D_2_ and 25(OH)D_3_ on the one hand and markers of low-grade inflammation and endothelial dysfunction on the other. However, associations between 25(OH)D_2_ and 25(OH)D_3_ and macroalbuminuria were independent of low-grade inflammation and endothelial dysfunction and may thus rather be mediated by processes other than inflammation and endothelial dysfunction, such as up-regulation of the RAAS [7, 10].

Conversion of 25(OH)D into its active form 1,25(OH)_2_D is reduced in individuals with decreased kidney function [1]. Therefore, eGFR may modify the associations between 25(OH)D_2_ and 25(OH)D_3_ on the one hand and microvascular complications and CVD on the other. However, we found no evidence to support this contention. This may be explained by the low number of individuals with impaired kidney function. Larger prospective studies that include individuals with a larger variety in eGFR are needed to fully address this issue. Similarly, parathyroid hormone stimulates the kidneys to produce 1,25(OH)_2_D [1] and may therefore also modify the association between vitamin D and vascular complications. In addition, magnesium has an essential role in the synthesis and metabolism of vitamin D and magnesium intake has already been shown to modify the association between vitamin D and risk of disease death in men [40]. Whether or not parathyroid hormone and/or magnesium intake modify the association between vitamin D and microvascular complications and CVD in type 1 diabetes needs to be further investigated.

### Strengths and limitations

Strengths of the current study include the measurement of both circulating 25(OH)D_2_ and 25(OH)D_3_. This is the first observational cohort study in individuals with type 1 diabetes investigating associations between 25(OH)D_2_ and vascular complications. In addition, we extensively controlled all associations for potential confounders including season of examination. Furthermore, plasma concentrations of vitamin D were comparable with those previously reported in other cohort studies in individuals with type 1 diabetes [11, 13], which indicates good data integrity of our data.

A drawback of the current study is its cross-sectional design that does not allow conclusions on causality regarding the associations between 25(OH)D_2_ and 25(OH)D_3_ and microvascular complications of diabetes. Furthermore, (cross-sectional) observational studies on vitamin D are particularly susceptible to reverse causation as any disease state is likely to reduce physical activity and thereby UV light exposure. However, we could confirm the associations between circulating 25(OH)D_3_ and albuminuria with similar associations between circulating 25(OH)D_2_ (which is not directly influenced by exposure to UV light) and albuminuria. In addition, although we have extensively adjusted for cardiovascular risk factors including physical activity, and, in the case of 25(OH)D_3_ for seasonal variation, residual confounding may still have occurred. In the current study we have no information on the use of vitamin D supplements, which is expected to be a determinant of vitamin D levels and may additionally represent health consciousness and healthy behaviour, which may be related to vascular disease. Last, even though data on the active vitamin D metabolite 1,25(OH)_2_D were not available for analyses, 25(OH)D concentrations may correlate better with clinical outcomes than 1,25(OH)_2_D concentrations as most cardiovascular and inflammatory cells express 1αhydroxylase enabling local synthesis of 1,25(OH)_2_D. Measurement of circulating 1,25(OH)_2_D does not include locally synthesized 1,25(OH)_2_D, whereas this local synthesis of 1,25(OH)_2_D seems particularly important in the non-skeletal actions of vitamin D [39, 41].

## Conclusion

In individuals with type 1 diabetes, both higher 25(OH)D_2_ and 25(OH)D_3_ are associated with a lower prevalence of macroalbuminuria, but not with retinopathy and CVD. These associations are independent of season of examination and known cardiovascular risk factors such as physical inactivity, and independent of low-grade inflammation and endothelial dysfunction. Larger and prospective studies are needed to further examine the associations between 25(OH)D_2_ and 25(OH)D_3_ and the development of microvascular complications and CVD in type 1 diabetes.

## Additional file

Additional file 1: Figure S1. Concentration of 25(OH)D_2_ (A) and 25(OH)D_3_ (B) according to latitude of the study centre. City, country code and, between brackets, latitude of the study centre, and the number of individuals per study centre are displayed on the x-axis. Boxes indicate medians and interquartile ranges and whiskers represent the minimum and maximum values. **Table S1.** Associations between 25-hydroxyvitamin D_2_ tertiles and prevalent albuminuria, retinopathy and cardiovascular disease. **Table S2.** Associations between month-specific 25-hydroxyvitamin D_3_ tertiles and prevalent albuminuria, retinopathy and CVD. **Table S3.** Associations between 25-hydroxyvitamin D_2_ levels and markers of low-grade inflammation and endothelial dysfunction. **Table S4.** Associations between 25-hydroxyvitamin D_3_ levels and markers of inflammation and endothelial dysfunction.

## References

[1] Holick MF (2007). Vitamin D, deficiency. N Engl J Med.

[2] Equils O, Naiki Y, Shapiro AM, Michelsen K, Lu D, Adams J (2006). 1,25-Dihydroxyvitamin D inhibits lipopolysaccharide-induced immune activation in human endothelial cells. Clin Exp Immunol.

[3] Schleithoff SS, Zittermann A, Tenderich G, Berthold HK, Stehle P, Koerfer R (2006). Vitamin D supplementation improves cytokine profiles in patients with congestive heart failure: a double-blind, randomized, placebo-controlled trial. Am J Clin Nutr.

[4] Al-Daghri NM, Guerini FR, Al-Attas OS, Alokail MS, Alkharfy KM, Draz HM (2014). Vitamin D receptor gene polymorphisms are associated with obesity and inflammosome activity. PLoS One.

[5] Molinari C, Uberti F, Grossini E, Vacca G, Carda S, Invernizzi M (2011). 1alpha,25-dihydroxycholecalciferol induces nitric oxide production in cultured endothelial cells. Cell Physiol Biochem.

[6] Sugden JA, Davies JI, Witham MD, Morris AD, Struthers AD (2008). Vitamin D improves endothelial function in patients with Type 2 diabetes mellitus and low vitamin D levels. Diabet Med.

[7] Li YC, Kong J, Wei M, Chen ZF, Liu SQ, Cao LP (2002). 1,25-Dihydroxyvitamin D(3) is a negative endocrine regulator of the renin-angiotensin system. J Clin Invest.

[8] Schram MT, Chaturvedi N, Schalkwijk CG, Fuller JH, Stehouwer CD (2005). Markers of inflammation are cross-sectionally associated with microvascular complications and cardiovascular disease in type 1 diabetes–the EURODIAB Prospective Complications Study. Diabetologia.

[9] Astrup AS, Tarnow L, Pietraszek L, Schalkwijk CG, Stehouwer CD, Parving HH (2008). Markers of endothelial dysfunction and inflammation in type 1 diabetic patients with or without diabetic nephropathy followed for 10 years: association with mortality and decline of glomerular filtration rate. Diabetes Care.

[10] Patel VB, Parajuli N, Oudit GY (2014). Role of angiotensin-converting enzyme 2 (ACE2) in diabetic cardiovascular complications. Clin Sci (Lond).

[11] de Boer IH, Sachs MC, Cleary PA, Hoofnagle AN, Lachin JM, Molitch ME (2012). Circulating vitamin D metabolites and kidney disease in type 1 diabetes. J Clin Endocrinol Metab.

[12] Kaur H, Donaghue KC, Chan AK, Benitez-Aguirre P, Hing S, Lloyd M (2011). Vitamin D deficiency is associated with retinopathy in children and adolescents with type 1 diabetes. Diabetes Care.

[13] Joergensen C, Hovind P, Schmedes A, Parving HH, Rossing P (2011). Vitamin D levels, microvascular complications, and mortality in type 1 diabetes. Diabetes Care.

[14] Beveridge LA, Witham MD (2013). Vitamin D and the cardiovascular system. Osteoporos Int.

[15] Cashman KD, Kinsella M, McNulty BA, Walton J, Gibney MJ, Flynn A (2014). Dietary vitamin D(2)–a potentially underestimated contributor to vitamin D nutritional status of adults?. Br J Nutr.

[16] Stephenson F, Fuller JH, on behalf of the EURODIAB IDDM Complications Study Group (1994). Microvascular and acute complications in IDDM patients: the EURODIAB IDDM Complications Study. Diabetologia.

[17] Koivisto VA, Stevens LK, Mattock M, Ebeling P, Muggeo M, Stephenson J (1996). Cardiovascular disease and its risk factors in IDDM in Europe. EURODIAB IDDM Complications Study Group. Diabetes Care.

[18] Chaturvedi N, Schalkwijk CG, Abrahamian H, Fuller JH, Stehouwer CD (2002). Circulating and urinary transforming growth factor beta1, Amadori albumin, and complications of type 1 diabetes: the EURODIAB prospective complications study. Diabetes Care.

[19] Chaturvedi N, Bandinelli S, Mangili R, Penno G, Rottiers RE, Fuller JH (2001). Microalbuminuria in type 1 diabetes: rates, risk factors and glycemic threshold. Kidney Int.

[20] Aldington SJ, Kohner EM, Meuer S, Klein R, Sjolie AK (1995). Methodology for retinal photography and assessment of diabetic retinopathy: the EURODIAB IDDM complications study. Diabetologia.

[21] Levey AS, Stevens LA, Schmid CH, Zhang YL, Castro AF, Feldman HI (2009). A new equation to estimate glomerular filtration rate. Ann Intern Med.

[22] Tielemans SM, Soedamah-Muthu SS, De Neve M, Toeller M, Chaturvedi N, Fuller JH (2013). Association of physical activity with all-cause mortality and incident and prevalent cardiovascular disease among patients with type 1 diabetes: the EURODIAB Prospective Complications Study. Diabetologia.

[23] Beulens JW, Kruidhof JS, Grobbee DE, Chaturvedi N, Fuller JH, Soedamah-Muthu SS (2008). Alcohol consumption and risk of microvascular complications in type 1 diabetes patients: the EURODIAB Prospective Complications Study. Diabetologia.

[24] Schram MT, Chaturvedi N, Schalkwijk C, Giorgino F, Ebeling P, Fuller JH (2003). Vascular risk factors and markers of endothelial function as determinants of inflammatory markers in type 1 diabetes: the EURODIAB Prospective Complications Study. Diabetes Care.

[25] Sterne JA, White IR, Carlin JB, Spratt M, Royston P, Kenward MG (2009). Multiple imputation for missing data in epidemiological and clinical research: potential and pitfalls. Bmj..

[26] Knol MJ, van der Tweel I, Grobbee DE, Numans ME, Geerlings MI (2007). Estimating interaction on an additive scale between continuous determinants in a logistic regression model. Int J Epidemiol.

[27] van Bussel BC, Soedamah-Muthu SS, Henry RM, Schalkwijk CG, Ferreira I, Chaturvedi N (2013). Unhealthy dietary patterns associated with inflammation and endothelial dysfunction in type 1 diabetes: the EURODIAB study. Nutr Metab Cardiovasc Dis.

[28] Jones G (2013). Extrarenal vitamin D activation and interactions between vitamin D(2), vitamin D(3), and vitamin D analogs. Annu Rev Nutr.

[29] O’Seaghdha CM, Hwang SJ, Holden R, Booth SL, Fox CS (2012). Phylloquinone and vitamin D status: associations with incident chronic kidney disease in the Framingham Offspring cohort. Am J Nephrol.

[30] Cai X, Hu Z, Chen L, Han X, Ji L (2014). Analysis of the associations between vitamin D and albuminuria or beta-cell function in Chinese type 2 diabetes. Biomed Res Int.

[31] Ahmadieh H, Azar ST, Lakkis N, Arabi A (2013). Hypovitaminosis d in patients with type 2 diabetes mellitus: a relation to disease control and complications. ISRN Endocrinol.

[32] Damasiewicz MJ, Magliano DJ, Daly RM, Gagnon C, Lu ZX, Ebeling PR (2012). 25-Hydroxyvitamin D levels and chronic kidney disease in the AusDiab (Australian Diabetes, Obesity and Lifestyle) study. BMC Nephrol.

[33] de Boer IH, Ioannou GN, Kestenbaum B, Brunzell JD, Weiss NS (2007). 25-Hydroxyvitamin D levels and albuminuria in the Third National Health and Nutrition Examination Survey (NHANES III). Am J Kidney Dis.

[34] Skaaby T, Husemoen LL, Pisinger C, Jorgensen T, Thuesen BH, Rasmussen K (2013). Vitamin D status and 5-year changes in urine albumin creatinine ratio and parathyroid hormone in a general population. Endocrine.

[35] Patrick PA, Visintainer PF, Shi Q, Weiss IA, Brand DA (2012). Vitamin D and retinopathy in adults with diabetes mellitus. Arch Ophthalmol.

[36] Payne JF, Ray R, Watson DG, Delille C, Rimler E, Cleveland J (2012). Vitamin D insufficiency in diabetic retinopathy. Endocr Pract.

[37] Theodoratou E, Tzoulaki I, Zgaga L, Ioannidis JP (2014). Vitamin D and multiple health outcomes: umbrella review of systematic reviews and meta-analyses of observational studies and randomised trials. BMJ.

[38] Lavie CJ, Lee JH, Milani RV (2011). Vitamin D and cardiovascular disease will it live up to its hype?. J Am Coll Cardiol.

[39] Norman PE, Powell JT (2014). Vitamin D and cardiovascular disease. Circ Res.

[40] Mursu J, Nurmi T, Voutilainen S, Tuomainen TP, Virtanen JK (2015). The association between serum 25-hydroxyvitamin D3 concentration and risk of disease death in men: modification by magnesium intake. Eur J Epidemiol.

[41] Mosekilde L (2008). Vitamin D, requirement and setting recommendation levels: long-term perspectives. Nutr Rev.

