# Supplementary material for: Low 25-hydroxyvitamin D2 and 25-hydroxyvitamin D3 levels are independently associated with macroalbuminuria, but not with retinopathy and macrovascular disease in type 1 diabetes: the EURODIAB prospective complications study
Source: Cardiovasc Diabetol. 2015 May 30;14:67. doi: 10.1186/s12933-015-0231-2 (PMC4456046; doi:10.1186/s12933-015-0231-2)
Supplement: Additional file 1: Figure S1. — Concentration of 25(OH)D2 (A) and 25(OH)D3 (B) according to latitude of the study centre. City, country code and, between brackets, latitude of the study centre, and the number of individuals per study centre are displayed on the x-axis. Boxes indicate medians and interquartile ranges and whiskers represent the minimum and maximum values. Table S1. Associations between 25-hydroxyvitamin D2 tertiles and prevalent albuminuria, retinopathy and cardiovascular disease. Table S2. Associations between month-specific 25-hydroxyvitamin D3 tertiles and prevalent albuminuria, retinopathy and CVD. Table S3. Associations between 25-hydroxyvitamin D2 levels and markers of low-grade inflammation and endothelial dysfunction. Table S4. Associations between 25-hydroxyvitamin D3 levels and markers of inflammation and endothelial dysfunction. [file 12933_2015_231_MOESM1_ESM.pdf]

Low 25-hydroxyvitamin D_2 and 25-hydroxyvitamin D_3 levels are independently associated with macroalbuminuria, but not with retinopathy and macrovascular disease in type 1 diabetes: the EURODIAB Prospective Complications Study

Lian Engelen, Casper G. Schalkwijk, Simone J.P.M. Eussen, Jean L.J.M. Scheijen, Sabita S. Soedamah-Muthu, Nish Chaturvedi, John H. Fuller and Coen D.A. Stehouwer

-Electronic Supplementary Material -

Additional file 1: Figure S1 Concentration of 25(OH)D_2 (**A**) and 25(OH)D_3 (**B**)according to latitude of the study centre. City, country code and, between brackets, latitude of the study centre, and the number of individuals per study centre are displayed on the x-axis. Boxes indicate medians and interquartile ranges and whiskers represent the minimum and maximum values.

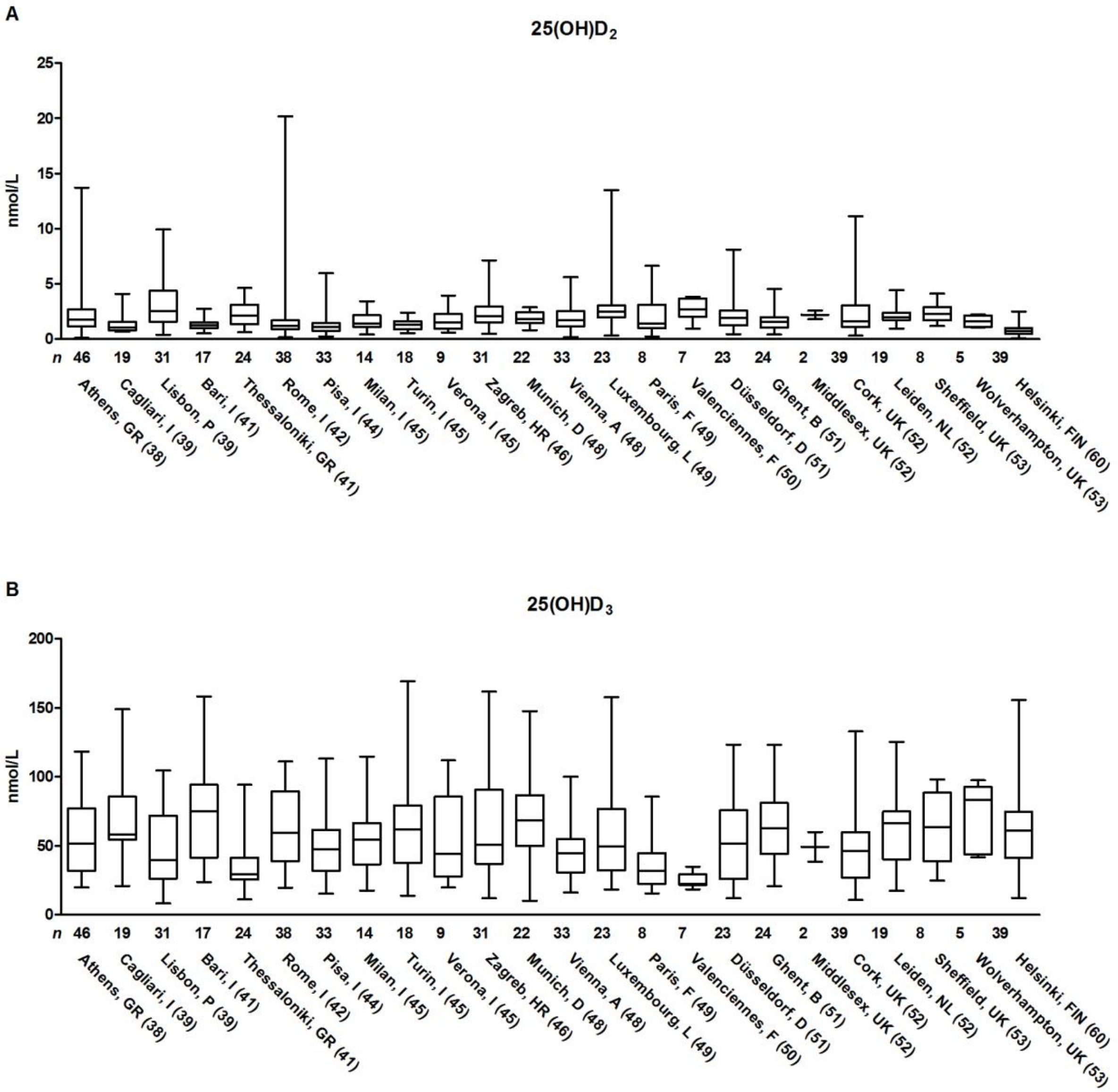

Figure S1

Table S1: Associations between 25-hydroxyvitamin D_2 tertiles and prevalent albuminuria, retinopathy and cardiovascular disease

		25-hydroxyvitamin D_2 tertiles						
		T1		T2		T3		*p-trend*
	Model	OR		OR	95% CI	OR	95% CI	
Microalbuminuria (n=83)	1	1.0	(reference)	0.87	0.47; 1.61	0.74	0.40; 1.37	0.34
	2	1.0	(reference)	0.86	0.41; 1.80	0.53	0.25; 1.12	0.09
	3	1.0	(reference)	0.92	0.44; 1.94	0.55	0.26; 1.17	0.10
	4	1.0	(reference)	0.94	0.44; 2.00	0.54	0.25; 1.15	0.09
Macroalbuminuria (n=124)	1	1.0	(reference)	0.29	0.18; 0.49	0.12	0.07; 0.23	<0.001
	2	1.0	(reference)	0.39	0.19; 0.80	0.13	0.06; 0.30	<0.001
	3	1.0	(reference)	0.41	0.20; 0.86	0.14	0.06; 0.31	<0.001
	4	1.0	(reference)	0.42	0.20; 0.86	0.13	0.06; 0.30	<0.001
Non-proliferative retinopathy (n=146)	1	1.0	(reference)	0.67	0.39; 1.13	0.60	0.35; 1.02	0.07
	2	1.0	(reference)	0.70	0.35; 1.39	0.95	0.48; 1.89	0.95
Proliferative retinopathy (n=152)	1	1.0	(reference)	0.46	0.27; 0.77	0.51	0.31; 0.85	0.009
	2	1.0	(reference)	0.73	0.34; 1.55	1.60	0.76; 3.37	0.16
CVD (n=131)	1	1.0	(reference)	1.88	1.11; 3.20	1.45	0.85; 2.49	0.18
	2	1.0	(reference)	2.22	1.23; 4.01	1.64	0.89; 3.05	0.13

OR, odds ratio indicates the odds of prevalent albuminuria, retinopathy and CVD, for 25-hydroxyvitamin D_2 tertile (T) 2 and T3 vs. T1. Model 1: adjusted for age and sex; Model 2: model 1 + BMI, smoking (never, ex, current), HbA_{1c}, total-HDL-cholesterol-ratio, systolic blood pressure, use of antihypertensive medication, eGFR, physical activity (0, ≤sex-specific median, >sex-specific median), alcohol intake (0, ≤70 g/wk, >70 g/wk), and prevalent CVD, albuminuria and retinopathy, as appropriate; Model 3: model 2 + low-grade inflammation score; Model 4: model 2 + endothelial dysfunction score.

Table S2: Associations between 25-hydroxyvitamin D_3 tertiles and prevalent albuminuria, retinopathy and CVD

		month-specific 25-hydroxyvitamin D_3 tertiles						
		T1		T2		T3		*p-trend*
	Model	OR		OR	95% CI	OR	95% CI	
Microalbuminuria (n=83)	1	1.0	(reference)	1.26	0.91; 1.71	0.65	0.35; 1.22	0.16
	2	1.0	(reference)	1.81	0.89; 3.69	0.80	0.38; 1.72	0.56
	3	1.0	(reference)	2.02	0.97; 4.18	0.88	0.41; 1.91	0.73
	4	1.0	(reference)	1.80	0.88; 3.67	0.81	0.38; 1.75	0.60
Macroalbuminuria (n=124)	1	1.0	(reference)	0.61	0.47; 0.79	0.29	0.17; 0.51	<0.001
	2	1.0	(reference)	0.97	0.48; 1.94	0.32	0.14; 0.69	0.005
	3	1.0	(reference)	1.06	0.52; 2.16	0.34	0.15; 0.75	0.008
	4	1.0	(reference)	0.97	0.48; 1.94	0.32	0.15; 0.72	0.006
Non-proliferative retinopathy (n=146)	1	1.0	(reference)	0.69	0.41; 1.17	0.43	0.25; 0.74	0.002
	2	1.0	(reference)	0.77	0.40; 1.49	0.71	0.36; 1.39	0.32
Proliferative retinopathy (n=152)	1	1.0	(reference)	0.70	0.42; 1.17	0.50	0.30; 0.84	0.008
	2	1.0	(reference)	0.93	0.45; 1.91	0.95	0.46; 1.98	0.91
CVD (n=131)	1	1.0	(reference)	1.07	0.65; 1.77	0.77	0.46; 1.31	0.36
	2	1.0	(reference)	1.23	0.71; 2.12	0.94	0.52; 1.70	0.86

OR, odds ratio indicates the odds of prevalent albuminuria, retinopathy and CVD for month-specific 25-hydroxyvitamin D_3 tertile (T) 2 and T3 vs. T1. Model 1: adjusted for age and sex; Model 2: model 1 + BMI, smoking (never, ex, current), HbA_{1c}, total-HDL-cholesterol-ratio, systolic blood pressure, use of antihypertensive medication, eGFR, physical activity (0, ≤sex-specific median, >sex-specific median), alcohol intake (0, ≤70 g/wk, >70 g/wk), and prevalent albuminuria, retinopathy and CVD, as appropriate; Model 3: model 2 + low-grade inflammation score; Model 4: model 2 + endothelial dysfunction score.

Table S3: Associations between 25-hydroxyvitamin D_2 levels and markers of low-grade inflammation and endothelial dysfunction

		25-hydroxyvitamin D_2 per 1 nmol/L			25-hydroxyvitamin D_2 tertiles						
					T1		T2		T3		*p-trend*
	Model	β	95% CI	*p*	β		β	95% CI	β	95% CI	
LGI score	1	-0.050	-0.094; -0.006	0.027	0.0	(reference)	-0.307	-0.500; -0.115	-0.325	-0.517; -0.132	0.001
	2	-0.026	-0.068; 0.015	0.22	0.0	(reference)	-0.239	-0.420; -0.058	-0.190	-0.371; -0.009	0.042
Ln(CRP)	1	-0.015	-0.061; 0.031	0.52	0.0	(reference)	-0.056	-0.256; 0.144	-0.066	-0.266; 0.134	0.52
	2	-0.005	-0.049; 0.039	0.82	0.0	(reference)	-0.078	-0.270; 0.114	-0.028	-0.221; 0.164	0.78
Ln(IL-6)	1	-0.013	-0.059; 0.034	0.60	0.0	(reference)	-0.211	-0.416; -0.005	-0.151	-0.355; 0.052	0.15
	2	-0.004	-0.051; 0.043	0.87	0.0	(reference)	-0.181	-0.386; 0.025	-0.093	-0.298; 0.112	0.38
Ln(TNF-α)	1	-0.081	-0.125; -0.038	<0.001	0.0	(reference)	-0.405	-0.594; -0.216	-0.493	-0.683; -0.303	<0.001
	2	-0.049	-0.087; -0.010	0.013	0.0	(reference)	-0.264	-0.432; -0.097	-0.294	-0.465; -0.124	0.001
ED score	1	-0.036	-0.082; 0.011	0.13	0.0	(reference)	-0.456	-0.658; -0.254	-0.355	-0.553; -0.156	0.001
	2	-0.016	-0.061; 0.028	0.48	0.0	(reference)	-0.363	-0.557; -0.170	-0.234	-0.426; -0.043	0.019
sE-selectin	1	0.012	-0.035; 0.058	0.62	0.0	(reference)	-0.262	-0.467; -0.058	-0.066	-0.269; 0.136	0.53
	2	0.009	-0.038; 0.055	0.72	0.0	(reference)	-0.249	-0.450; -0.048	-0.060	-0.260; 0.140	0.58
sVCAM-1	1	-0.064	-0.111; -0.017	0.008	0.0	(reference)	-0.397	-0.601; -0.193	-0.446	-0.648; -0.244	<0.001
	2	-0.032	-0.076; 0.012	0.16	0.0	(reference)	-0.276	-0.467; -0.084	-0.279	-0.469; -0.088	0.004

β, regression coefficient indicates the increase in markers of inflammation and endothelial dysfunction (in SD) per 1 nmol/L higher 25-hydroxyvitamin D_2 (first column) or 25-hydroxyvitamin D_2 tertile (T) 2 and T3 vs. T1 (last three columns). Model 1: adjusted for age, sex and case-control status; Model 2: model 1 + BMI, smoking (never, ex, current), HbA_{1c}, total-HDL-cholesterol-ratio, systolic blood pressure, use of antihypertensive medication, eGFR, physical activity (0, ≤sex-specific median, >sex-specific median), alcohol intake (0, ≤70 g/wk, >70 g/wk). *LGI*, low-grade inflammation; *ED*, endothelial dysfunction.

Table S4: Associations between 25-hydroxyvitamin D_3 levels and markers of inflammation and endothelial dysfunction

		25-hydroxyvitamin D_3 per 10 nmol/L			month-specific 25-hydroxyvitamin D_3 tertiles						
					T1		T2		T3		*p-trend*
	Model	β	95% CI	*p*	β		β	95% CI	β	95% CI	
LGI score	1	-0.034	-0.066; -0.003	0.032	0.0	(reference)	-0.287	-0.480; -0.093	-0.215	-0.410; -0.020	0.031
	2	-0.019	-0.049; 0.011	0.21	0.0	(reference)	-0.206	-0.387; -0.024	-0.131	-0.317; 0.056	0.17
Ln(CRP)	1	-0.016	-0.048; 0.017	0.35	0.0	(reference)	-0.202	-0.402; -0.002	-0.122	-0.325; 0.081	0.24
	2	-0.010	-0.041; 0.022	0.55	0.0	(reference)	-0.165	-0.357; 0.026	-0.091	-0.288; 0.107	0.37
Ln(IL-6)	1	-0.029	-0.063; 0.004	0.09	0.0	(reference)	-0.275	-0.480; -0.070	-0.249	-0.456; -0.042	0.018
	2	-0.017	-0.051; 0.018	0.35	0.0	(reference)	-0.219	-0.426; -0.012	-0.175	-0.388; 0.037	0.10
Ln(TNF-α)	1	-0.030	-0.066; 0.002	0.07	0.0	(reference)	-0.151	-0.345; 0.044	-0.098	-0.294; 0.098	0.33
	2	-0.016	-0.045; 0.013	0.29	0.0	(reference)	-0.066	-0.238; 0.106	-0.020	-0.197; 0.157	0.83
ED score	1	-0.048	-0.082; -0.015	0.005	0.0	(reference)	-0.205	-0.407; -0.002	-0.272	-0.478; -0.066	0.010
	2	-0.040	-0.073; -0.007	0.017	0.0	(reference)	-0.149	-0.343; 0.046	-0.215	-0.416; -0.015	0.035
sE-selectin	1	-0.022	-0.056; 0.012	0.20	0.0	(reference)	-0.037	-0.242; 0.168	-0.155	-0.363; 0.053	0.14
	2	-0.020	-0.054; 0.014	0.24	0.0	(reference)	-0.002	-0.203; 0.200	-0.128	-0.336; 0.080	0.23
sVCAM-1	1	-0.048	-0.082; -0.014	0.006	0.0	(reference)	-0.258	-0.463; -0.053	-0.238	-0.446; -0.029	0.025
	2	-0.037	-0.069; -0.005	0.024	0.0	(reference)	-0.213	-0.404; -0.021	-0.183	-0.381; 0.015	0.07

β, regression coefficient indicates the increase in markers of inflammation and endothelial dysfunction (in SD) per 10 nmol/L higher 25-hydroxyvitamin D_3 (first column) or month-specific 25-hydroxyvitamin D_3 tertile (T) 2 and T3 vs. T1 (last three columns). Model 1: adjusted for age, sex and case-control status (and, in the analyses with continuous 25-hydroxyvitamin D_3 levels, season); Model 2: model 1 + BMI, smoking (never, ex, current), HbA_{1c}, total-HDL-cholesterol-ratio, systolic blood pressure, use of antihypertensive medication, eGFR, physical activity (0, ≤sex-specific median, >sex-specific median), alcohol intake (0, ≤70 g/wk, >70 g/wk).

The EURODIAB Prospective Complications Study Group

B. Karamanos, A. Kofinis, K. Petrou, Hippokration Hospital, Athens, Greece; F. Giorgino, G. Picca, A. Angarano, G. De Pergola, L. Laviola, R. Giorgino, Internal Medicine, Endocrinology and Metabolic Diseases, Department of Emergency and Organ Transplantation, University of Bari, Bari, Italy; C. Ionescu-Tirgoviste, A. Coszma, C. Guja, Clinic of Diabetes, Nutrition and Metabolic Diseases, Bucharest, Romania; M. Songini, A. Casu, M. Pedron, S. Pintus, M. Fossarello, Diabetes Unit Ospedale San Michele, Cagliari, Italy; J. B. Ferriss, G. Grealy, D. O'Keefe, Cork University Hospital, Cork, Ireland; M. Toeller, C. Arden, Diabetes Research Institute, Heinrich-Heine University, Duesseldorf, Germany; R. Rottiers, C. Tuyttens, H. Priem, University Hospital of Gent, Belgium; P. Ebeling, M. Kylliäinen , V. A. Koivisto, University Hospital of Helsinki, Finland; B. Idzior-Walus, J. Sieradzki, K. Cyganek, B. Solnica, Department of Metabolic Diseases, Jagiellonian University, Krakow, Poland; H. H. P. J. Lemkes, J. C. Lemkes-Stuffken, Leiden University Medical Centre, the Netherlands; J. Nunes-Correa, M. C. Rogado, L. Gardete-Correia, M. C. Cardoso, A. Silva, J. Boavida, M. Machado Sa Marques, Portuguese Diabetic Association, Lisbon, Portugal; G. Michel, R. Wirion, S. Cardillo, Centre Hospitalier, Luxembourg; G. Pozza, R. Mangili, V. Asnaghi, Ospedale San Raffaele, Milan, Italy; E. Standl, B. Schaffler, H. Brand, A. Harms, City Hospital Schwabing, Munich, Germany; D. Ben Soussan, O. Verier-Mine, P. Fallas, M. C. Fallas , Centre Hospitalier de Valenciennes, France; J. H. Fuller, J. Holloway, L. Asbury, D. J. Betteridge, University College London, UK; G. Cathelineau, A. Bouallouche, B. Villatte Cathelineau, Hospital Saint-Louis, Paris, France; F. Santeusanio, G. Rosi, V. D'Alessandro, C. Cagini, P. Bottini, G. P. Reboldi, Dipartimento di Medicina Interna, Perugia, Italy; R. Navalesi, G. Penno, S. Bandinelli, R. Miccoli, M. Nannipieri, Dipartimento di Endocrinologia e Metabolismo, Pisa, Italy; G. Ghirlanda, C. Saponara, P. Cotroneo, A. Manto, A. Minnella, Universita Cattolica del Sacro Cuore, Rome, Italy; J. D. Ward, S. Tesfaye, S. Eaton, C. Mody, Royal Hallamshire Hospital, Sheffield, UK; M. Borra, P. Cavallo Perin, S. Giunti, G. Grassi, G. F. Pagano, M. Porta, R. Sivieri, F. Vitelli, M. Veglio, Dipartimento di Medicina Interna, Università di Torino and ASO TO/CRF/Maria Adelaide, Turin, Italy; N. Papazoglou, G. Manes, General Hospital Papageorgiou, Diabetes

Unit, Thessaloniki, Greece; M. Muggeo, M. Iagulli, V. Cacciatori, V. Cattedra di Malattie del Metabolismo, Verona, Italy; K. Irsigler, H. Abrahamian, Hospital Vienna Lainz, Austria; S. Walford, J. Sinclair, S. Hughes, V. McLelland, J. Ward, New Cross Hospital, Wolverhampton, UK; G. Roglic, Z. Metelko, Z. R. Pepeonik, Vuk Vrhovac Institute for Diabetes, Zagreb, Croatia

Steering committee members

J. H. Fuller (London), B. Karamanos, Chairman (Athens), A.-K. Sjolie (Odense), N. Chaturvedi (London), M. Toeller (Duesseldorf), G. Pozza Co-chairman (Milan), B. Ferriss (Cork), M. Porta (Turin), R. Rottiers (Gent), G. Michel (Luxembourg)

Co-ordinating centre

J. H. Fuller, N. Chaturvedi, J. Holloway, D. Webb, L. Asbury, University College London, UK

Central laboratories

G.-C. Viberti, R. Swaminathan, P. Lumb, A. Collins, S. Sankaralingham, M.A. Crook, Guy's and St Thomas Hospital, London, UK

Retinopathy Grading Centre

S. Aldington, T. Mortemore, H. Lipinski, Royal Postgraduate Medical School of Imperial College London, London, UK

Nutrition Co-ordinating Centre

M. Toeller, W.A. Scherbaum, F.A. Gries, Heinrich-Heine-University, Diabetes Research Institute and Department of Endocrinology, Diabetology and Rheumatology, Duesseldorf, Germany
